# Microstructural Characterisation of Austenitic Heat Resistant Sanicro 25 Steel after Steam Oxidation

**DOI:** 10.3390/ma13153382

**Published:** 2020-07-30

**Authors:** Bogdan Rutkowski

**Affiliations:** Faculty of Metals Engineering and Industrial Computer Science, AGH University of Science and Technology, Al. A. Mickiewicza 30, 30-059 Kraków, Poland; rutkowsk@agh.edu.pl

**Keywords:** steam oxidation, HRSTEM, steel, SEM

## Abstract

Microstructural and morphological observations of the surface scale on a high Cr and Ni austenitic heat resistant steel oxidised in water vapour at 700 °C are reported. Analysis of microstructure was carried out by analytical techniques of transmission- and scanning electron microscopy. Investigation of M_23_C_6_ nucleated at the interface between matrix and the Z-phase precipitates after exposing to high temperature showed semicoherency between M_23_C_6_ and the matrix and no coherency with the Z-phase. Plates developed on the oxide scale surface consist of Cr_2_O_3_ crystals separated by amorphous SiO_2_.

## 1. Introduction

In high efficiency conventional power stations, less coal combustion takes place as to obtain higher amount of energy in comparison to older generation power plants. This is the result of increases in the operating pressure and temperature of steam [1]. Well-known, used for decades, heat- and oxidation-resistant steels, however, suffer from serious microstructural instability problems at elevated temperatures. Stable microstructure at high operating temperature and long operation are a warranty of long-term working without malfunction, which avoids huge costs of down time of the power plant. Operation e.g., only 50 °C higher than 650 °C is not practical for conventional steels, since the elevated temperature causes strengthening precipitates to dissolve or coagulate, which decreases strength and leads to faster creep damage [2]. Moreover, at higher temperature, corrosion processes will take place faster or the corrosion mechanism might change, since various oxides have different free energies of formation [3]. This might lead to failure due to a decrease of the tube wall thickness. Therefore, increasing steam parameters forces engineers to develop new materials capable of withstanding the more severe conditions. Whereas steels with 12% Cr content are not suitable for applications above 650 °C [4], a high Cr and Ni content steel Sanicro 25 (22Cr25NiWCoCu) possesses increased corrosion resistance at high temperatures [5]. Nickel stabilises the austenite and strengthens the steel. Tungsten contributes to solid solution strengthening, similarly to P92 steel in comparison with the P91 grade [6,7]. Cu further strengthens Sanicro 25 [8], as small, 20–50 nm in size, ε-Cu precipitates, observed after exposure to high temperatures [9].

Current work is focused on microstructural characterisation of Sanicro 25 steel after steam exposure at 700 °C for up to 15,000 h. Various techniques, mainly high-resolution scanning transmission electron microscopy (HRSTEM), scanning electron microscopy (SEM) with low accelerating voltage and energy dispersive x-ray spectroscopy (EDS) were used for complex structural analysis.

## 2. Materials and Methods

The chemical composition of Sandvik Sanicro 25 is shown in Table 1.

Small (20 × 15 × 2 mm) coupons were cut from the as-received rod. In order to accommodate them in the furnace chamber, 1.5-mm diameter holes were drilled in each of the coupons. They were ground on abrasive papers with increasing gradation (from 600 to 4000 grit) and polished with diamond pastes (3, 1, 1/4 µm). Afterwards the samples were oxidised at 700 °C for up to 15,000 h. Test setup is described elsewhere [9,10]. Investigations on oxidized samples were performed both on sample surfaces and metallographic cross-sections. In order to prepare these, sample surfaces were coated with gold to assure electrical contact and then electroplated with pure nickel to protect the oxide scale during subsequent cutting and grinding. Afterwards the samples were cold embedded in epoxy resin, ground on abrasive papers (600–2500 grit) and polished on diamond suspensions (3, 1, 1/4 µm). The cross-sections were finally sputtered with thin (~2 nm) gold layer. 

**Figure 1:**
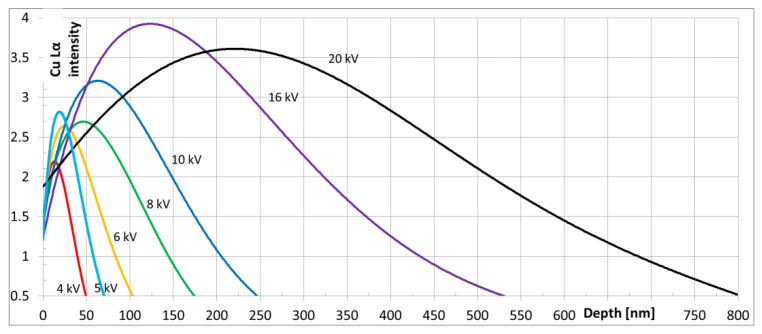
Results of calculation, showing depth of Lα X-ray emission of Cu.

Scanning electron microscope (Merlin of Zeiss, Oberkochen, Germany) was used to determine the chemical composition of the base material and microstructure, chemical composition and topography of the oxide scales. Since precipitates in Sanicro 25 can have a size of around 50–80 nm, the low voltage SEM-EDS technique was used in order to visualise them on elemental maps. Figure 1 illustrates the intensity of Cu-Lα line (0.928 keV), on the basis of Casino V2.51 software simulation (Université de Sherbrooke, Sherbrooke, QC, Canada) [11], used to visualise the Cu-containing precipitates. Rather than the ‘standard’ accelerating voltage of 20 kV, which would render such small particles ‘invisible’, 5 kV was used, such that the strongest yield of Cu-Lα signal was emitted from the depth of around 50 nm (FWHM).

Samples for transmission electron microscopy (TEM) were prepared by focus ion beam (FIB) milling with the final thinning performed with Nanomill 1040 (E.A. Fischione Instruments, Export, PA, USA). Specimens were examined on a probe Cs-corrected Titan^3^ G2 60–300 instrument (Thermo Fisher Scientific, Eindhoven, Netherlands). HRSTEM results were supported by simulations done with JEMS software (JEMS-SWISS, Jongny, Switzerland) [12].

## 3. Results and Discussion

### 3.1. As-Received Material

**Figure 2:**
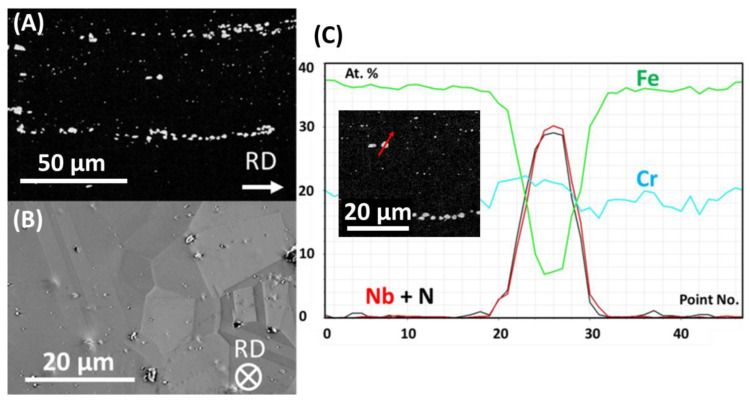
Microstructure of as-received Sanicro 25 steel: (**A**) longitudinal cross section, SEM-SE (contrast was enhanced to make primary precipitates very well visible), (**B**) transversal cross-section, SEM-SE, (**C**) results of SEM-EDS line scan along red arrow shown in the inset.

Microstructure of as-received sample in the rolling direction is shown in Figure 2A and in transverse to the rolling direction—in Figure 2B. The structure is typical of a supersaturated steel. Primary bands of NbCrN precipitates along the rolling direction (RD), crystallised from the liquid during solidifying, are present. Typical twinned structure of austenite grains is visible. Grain boundaries are free from precipitates. In Figure 2C, inset, SEM-EDS line scan along red arrow going across primary precipitate is shown. It is clearly visible that the matrix of the alloy is enriched in iron and chromium (approx. 40%, 20%, respectively). In the precipitate, however, Fe content is strongly decreased, chromium is maintained at the similar level as in the matrix and Nb and N contents are increased. That the primary precipitates contain Cr, Nb and N suggests tetragonal CrNbN, incoherent with the matrix. This was previously reported by electron diffraction results [13,14]. In lower Cr-content steels, instead of NbCrN, the matrix-coherent NbN with cubic cF8 structure is nucleated.

### 3.2. After Oxidation

**Figure 3:**
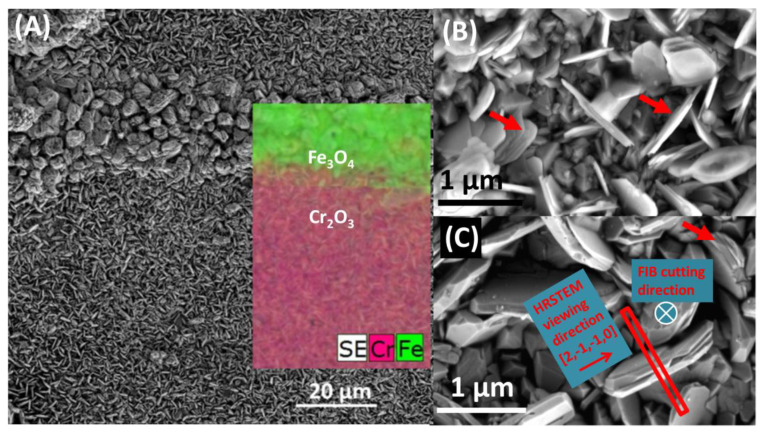
Morphology of oxide scale, grown on Sanicro 25 after oxidation at 700 °C (SEM-SE): (**A**) an overview of the surface after 500 h of oxidation. In the inset, the SE image was overlaid on the EDS results to enhance contrast of the elemental map, (**B**) plates after 500 h of oxidation at higher magnification, (**C**) plates after 10,000 h of oxidation at higher magnification. Overlays showing the direction of focus ion beam (FIB) lamella cut.

X-ray diffraction (XRD) measurements in Bragg-Brentano geometry [9], suggest the presence of two oxide phases Cr_2_O_3_ and (Fe,X)_3_O_4_, where X = Fe or Cr, clarified by SEM, Figure 3, showing the morphology of the oxide scale surface to be a mixture of two types of structures. Very thin plates are visible in most of the area.

SEM–EDS (inset in Figure 3A) revealed presence of Cr and nodules consisting of bigger crystals, enriched with Fe. It appears that oxide scale is continuous, without traces of spallation, suggesting very good adhesion to the substrate and therefore good protection against fast oxidation.

Figure 3B,C shows the oxide scale after 500 and 10,000 h, respectively, at a higher magnification. After the shorter time, the plates are very thin, subsequently much thicker plates, built up from few thinner ones, are formed. Characteristic growth terraces are present (marked with red arrows in Figure 3B,C). Moreover, every single plate of such composite is thicker than plates after 500 h of oxidation. Red rectangle in Figure 3C represents the FIB lamella, which was cut in a direction perpendicular to the large surface of the plate. Such a lamella was further investigated with TEM and HR(S)TEM indicated viewing direction.

**Figure 4:**
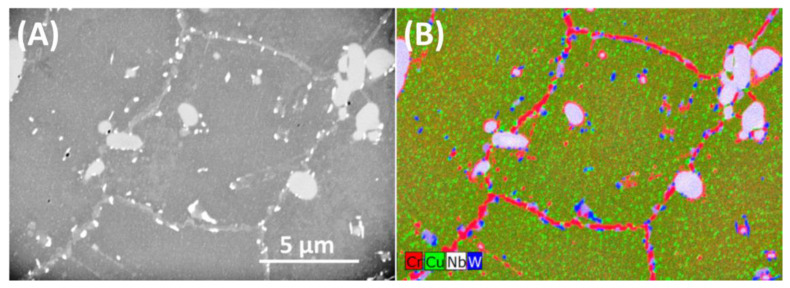
Microstructure of bulk material after oxidation at 700 °C for 5000 h: (**A**) SEM-SE image, (**B**) SEM-EDS elemental map of selected elements.

Figure 4A shows the microstructure of an area of oxidized sample on a metallographic cross-section a few millimetres from the sample surface. Therefore, this area has the nominal chemical composition and is not affected by corrosion.

Low-voltage EDS enabled observation of small (around 50 nm diameter), evenly distributed ε-Cu precipitates (green in Figure 4B). Elongated precipitates around 2 µm, smaller, round or elongated precipitates, all with bright contrast, and grain boundaries decorated with chains of precipitates (grey contrast) are visible. Results of low-voltage EDS, Figure 4B, show that the bigger elongated precipitates contain Nb, N, Cr, (in Figure 4B signal from N was omitted for sake of clarity). Around the bigger Z-phase particles, also smaller precipitates, containing mainly Cr and C were detected. Their chemical composition is similar to that of the continuous chain of precipitates at grain boundaries and suggests M_23_C_6_ in both cases. Small bright precipitates in Figure 4A are enriched in W and the regions in the immediate vicinity (~2 µm) of grain boundaries are depleted in Cr.

**Figure 5:**
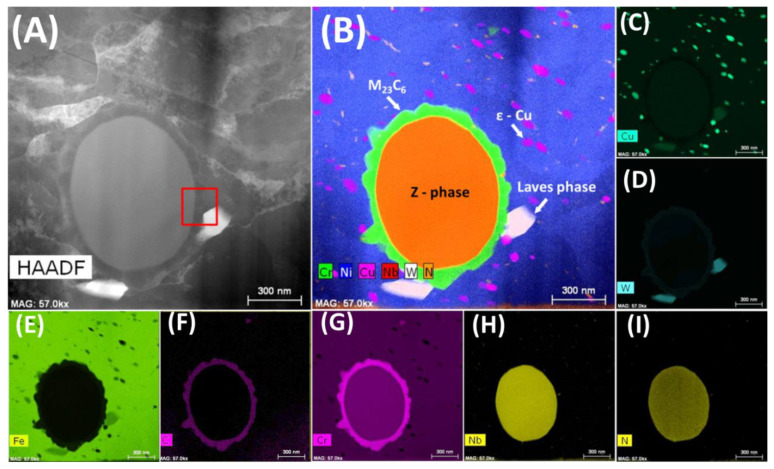
STEM data for the bulk material after 5000 h of oxidation at 700 °C: (**A**) HAADF image showing Z-phase, (**B**)–(**I**) EDS map of selected elements.

Figure 5A, acquired at a higher magnification using the STEM-HAADF (high-angle annular dark-field) technique, shows part of bulk material, where bigger precipitate of primary Z-phase is encapsulated by those of M_23_C_6_, nucleated on the austenite/Z-phase interface.

STEM-EDS results (Figure 5B–I) clearly indicate the presence of Cr, Nb, and N with 1:1 Cr to Nb ratio for Z-phase. The M_23_C_6_ precipitates contain mainly Cr, with a minor amount of W. White precipitates on Figure 5B consist of W, Fe, Cr, and Cu with stoichiometry close to (Fe,Cr,Cu)_2_W. Suo et al. also found Laves phase in Sanicro 25 after creep tests [15] and proposed that the precipitation is promoted by stress. ε-Cu precipitates, having the unit cell similar to that of austenite, consist of pure Cu and coagulate with time [9]. These small precipitates are desirable, to take part in the Orowan strengthening mechanism. Results of 300 kV STEM-EDS are consistent with the data obtained with low voltage SEM-EDS and show that the nm-sized ε-Cu precipitates can be observed by SEM-EDS.

**Figure 6:**
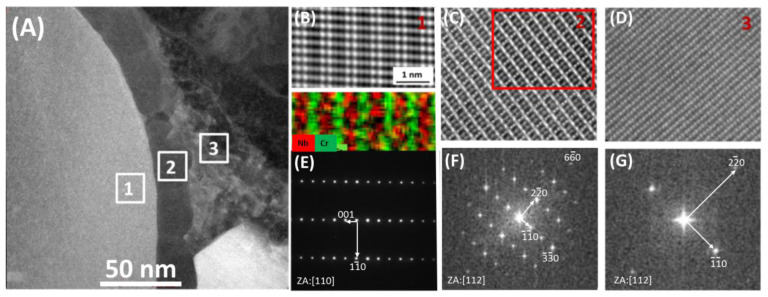
Area marked with red square in Figure 5A at a higher magnification: (**A**) HAADF image of Z-phase/M_23_C_6_/matrix interface, (**B**) HRSTEM image of Z-phase with EDS map (area 1 in Figure 6A), (**C**) HRSTEM image of M_23_C_6_ precipitate (area 2 in Figure 6A), (**D**) HRSTEM images of matrix (area 3 in Figure 6A), (**E**) SAED from the Z-phase, (**F**) FFT showing spots from M_23_C_6_, (**G**) FFT showing spots from the matrix.

HRSTEM investigation proved that M_23_C_6_ nucleates on the austenite side and is semicoherent with the matrix. The evidence is shown in Figure 6F,G, where fast Fourier transformed (FFT) images of austenite and M_23_C_6_ from areas #2 and #3, marked with small white squares on Figure 6A are presented. FFT images showing that every third reflection of M_23_C_6_ is overlapping with every first reflection of austenite {e.g., (6,-6,0)M_23_C_6_ and (2,-2,0)γ}, showing that the phases are semicoherent and that the interplanar distance of matrix is 1/3 of the interplanar distance of M_23_C_6_. Such relation between M_23_C_6_ and the matrix is well-known for steels and Ni-based alloys [16]. If the M_23_C_6_ precipitates were coherent with the matrix only, the Z-phase could act as a stress concentrator. It was found that the higher volume fraction of Z-phase after several thousand hours of creep contributes to a decrease of creep strength [17]. Moreover, higher Cr-content in the steel generates higher amount of Z-phase during annealing [18], while MX precipitates are dissolving. Therefore Z-phase is more stable at high temperature than MX precipitates. Sanicro 25 contains around 22% of Cr, making the presence of CrNbN even in the as-received because of the crystallization from the liquid during solidification.

Figure 6B shows high resolution image of the ordered structure of the Z-phase in [110] zone axis. Two rows of atoms with brighter contrast and two rows of atoms with darker contrast intertwine with each other. It suggests a 1:1 ratio of Nb to Cr atoms since the HAADF technique, which was used for imaging, gives contrast depending on the Z number of the element. Heavier elements are brighter. Therefore, brighter atoms are Nb, whereas darker contrast atoms are Cr, which was also confirmed by EDS (Figure 6B).

**Figure 7:**
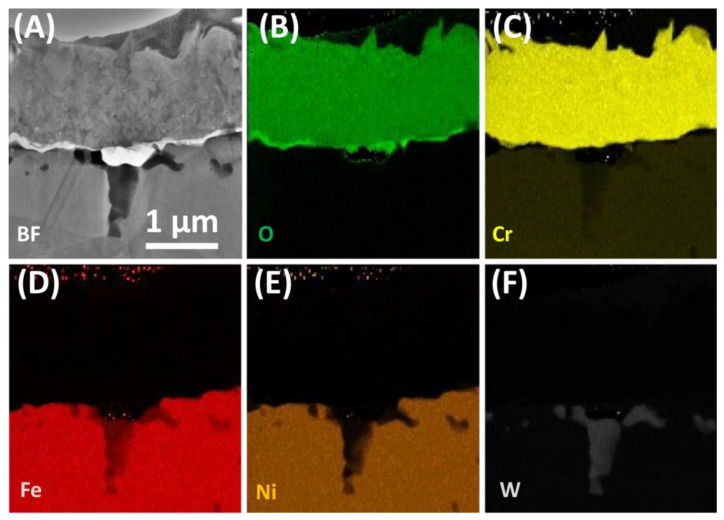
Oxide scale, grown on Sanicro 25 surface after 500 h of oxidation in steam at 700 °C: (**A**) STEM-BF image, (**B**)–(**F**) STEM-EDS elemental maps of selected elements.

General appearance of the protective Cr_2_O_3_ scale formed is shown in Figure 7A. Chemical composition of the oxide scale is homogenous and no trace of other phases is visible. Outward diffusion of chromium implied the existence of Cr-depleted area at the subsurface. Lowered amount of Cr in the subsurface is one of the reasons for the dissolution of M_23_C_6_ precipitates in the Cr-depleted region. The other reason is inward diffusion of carbon into the alloy due to a higher activity of C after partial dissolution of carbides, which further accelerates the process [19]. Decarburization of the alloy surface is another proposed reason for carbide dissolution [20]. Since general microstructure of developed oxide is known [21], emphasis was put on a detailed examination of the structure of the plates. Similar plates were observed by Webb and Forgeng on aluminium oxidized in wet hydrogen atmosphere [22], who reported the formation of volatile AlO compound and its further decomposition on the sample surface, where surface diffusion processes take place. Similar process may occur in chromium containing steels oxidised in steam forming gaseous CrO_2_(OH)_2_ [23].

**Figure 8:**
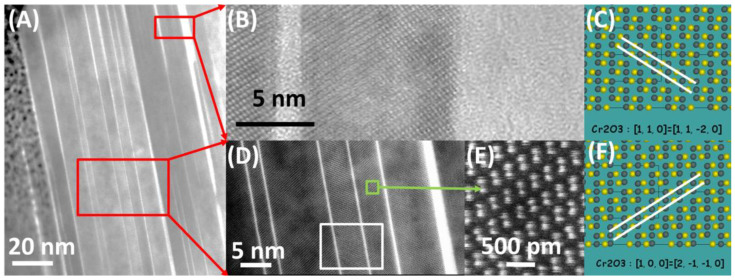
One plate of Cr_2_O_3_ oxide scale at a high magnification: (**A**) Overview showing sandwich-like structure (STEM-BF); (**B**) HRTEM image showing amorphous structure between Cr_2_O_3_ crystals; (**C**), (**F**) Cr_2_O_3_ unit cell drawing, generated with JEMS software for two different zone axes; (**D**) few crystals at higher magnification (STEM-BF); (**E**) HRSTEM-HAADF image of Cr_2_O_3_ crystal.

**Figure 9:**
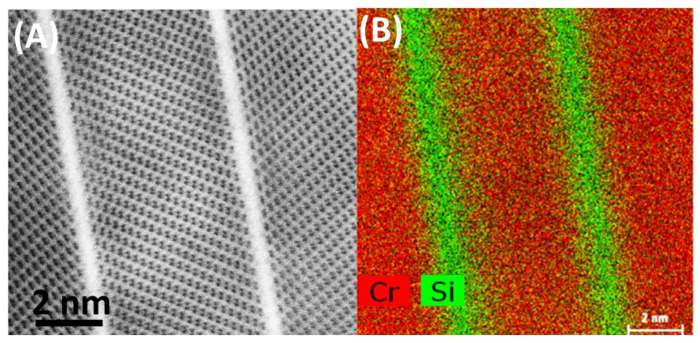
Cr_2_O_3_ crystals: (**A**) STEM-BF image, (**B**) STEM-EDS map of Cr and Si.

Figure 8A shows a typical cross-section of a plate. Consistent with the SEM image (Figure 3B,C), each of the bigger plates of the oxide scale is built of many thinner Cr_2_O_3_ crystals (light grey contrast), ‘bound’ together with a thin (few inter-atomic distances) layer, giving bright contrast. Results of further HR(S)TEM investigation are presented in Figure 8B,D,E. The section (D) shows an area of few Cr_2_O_3_ crystals growing in two directions. Further analysis with JEMS software revealed Cr_2_O_3_ plates having $\left[2\bar{1}\bar{1}0\right]$ and $\left[11\bar{2}0\right]$ zone axes. Figure 8E shows a high-resolution STEM-HAADF image of heavy Cr atoms in bright contrast. Light oxygen atoms are invisible. Drawing of atoms (Cr—yellow, O—grey) in Cr_2_O_3_ unit cell in the above-mentioned zone axes, generated with JEMS are shown in Figure 8C,F. White lines are guide for eyes. Cr atom positions fit perfectly to the HRSTEM image, proving the crystallographic orientation. Since the cross-section was cut across the largest surface of the plate, according to the geometry shown in Figure 3C, it can be concluded that large surfaces of Cr_2_O_3_ plates are (0001) planes. Areas between Cr_2_O_3_ crystals (white contrast in Figure 8A,D) are amorphous, evidenced in Figure 8B, acquired with HRTEM. Typical glass phase is visible between ordered structure of Cr_2_O_3_. Results of STEM-EDS investigation (Figure 9) on the area indicated with white rectangle in Figure 8D, revealed presence of silicon and oxygen between Cr_2_O_3_ crystals.

Presence of glassy SiO_2_ in oxide scale of austenitic steel was found by Rutkowski et al. [24] and later confirmed on other steel grades by Yoo et al. [25]. These oxide scales grown under fireside corrosion conditions were more complex, with glassy SiO_2_ phase in coexistence with few sublayers of other oxides. Here composite-like structure with Cr_2_O_3_ seems to have formed. It might be possible that diffusing from matrix, Si is oxidized at the surface of Cr_2_O_3_ plate, creating thin SiO_2_, on which another Cr_2_O_3_ plate grows. This mechanism is supported by grain boundaries being a fast path of Cr and Si diffusion, confirmed by finding SiO_2_ and Cr_2_O_3_ phases in the oxide scale above grain boundaries of the alloy [24].

## 4. Conclusions

1. During heat treatment of Sanicro 25 alloy, the matrix semicoherent M_23_C_6_ carbides nucleate on the interface between the matrix and the Z-phase,
2. Two-phase oxide scale developed on the steel surface is continuous and well- protective.
3. Characteristic plates of Cr_2_O_3_ are present, indicating the presence of volatile compounds decomposing at the surface.
4. Growth terraces are present at the surfaces of plates, suggesting the presence of surface diffusion processes.
5. Plates are built of Cr_2_O_3_ crystals, separated by amorphous SiO_2_ phase.

## Figures and Tables

**Table 1:** Chemical composition of Sanicro 25.

Element	Fe	Ni	Cr	W	Cu	Co	Mn	Nb	N	Si	C	Other
wt.%	42.8	25.4	22.4	3.4	3	1.5	0.5	0.5	0.2	0.2	0.06	0.04
